# Clinical evaluation of commercial nucleic acid amplification tests in patients with suspected sepsis

**DOI:** 10.1186/s12879-015-0938-4

**Published:** 2015-04-28

**Authors:** Lars Ljungström, Helena Enroth, Berndt EB Claesson, Ida Ovemyr, Jesper Karlsson, Berit Fröberg, Anna-Karin Brodin, Anna-Karin Pernestig, Gunnar Jacobsson, Rune Andersson, Diana Karlsson

**Affiliations:** Department of Infectious Diseases, Skaraborg Hospital, SE-541 85 Skövde, Sweden; Department of Clinical Microbiology, Unilabs AB, SE-541 85 Skövde, Sweden; Systems Biology Research Centre, School of Biosciences, University of Skövde, SE-541 28 Skövde, Sweden; Institute of Biomedicine, Sahlgrenska Academy, Gothenburg University, SE-402 34 Gothenburg, Sweden

**Keywords:** Blood culture, Magicplex™, Prove-it™, sepsis diagnostic, multiplex PCR, microarray

## Abstract

**Background:**

Sepsis is a serious medical condition requiring timely administered, appropriate antibiotic therapy. Blood culture is regarded as the gold standard for aetiological diagnosis of sepsis, but it suffers from low sensitivity and long turnaround time. Thus, nucleic acid amplification tests (NAATs) have emerged to shorten the time to identification of causative microbes. The aim of the present study was to evaluate the clinical utility in everyday practice in the emergency department of two commercial NAATs in patients suspected with sepsis.

**Methods:**

During a six-week period, blood samples were collected consecutively from all adult patients admitted to the general emergency department for suspicion of a community-onset sepsis and treated with intravenous antibiotics. Along with conventional blood cultures, multiplex PCR (Magicplex™) was performed on whole blood specimens whereas portions from blood culture bottles were used for analysis by microarray-based assay (Prove-it™). The aetiological significance of identified organisms was determined by two infectious disease physicians based on clinical presentation and expected pathogenicity.

**Results:**

Among 382 episodes of suspected sepsis, clinically relevant microbes were detected by blood culture in 42 episodes (11%), by multiplex PCR in 37 episodes (9.7%), and by microarray in 32 episodes (8.4%). Although moderate agreement with blood culture (kappa 0.50), the multiplex PCR added diagnostic value by timely detection of 15 clinically relevant findings in blood culture-negative specimens. Results of the microarray corresponded very well to those of blood culture (kappa 0.90), but were available just marginally prior to blood culture results.

**Conclusions:**

The use of NAATs on whole blood specimens in adjunct to current culture-based methods provides a clinical add-on value by allowing for detection of organisms missed by blood culture. However, the aetiological significance of findings detected by NAATs should be interpreted with caution as the high analytical sensitivity may add findings that do not necessarily corroborate with the clinical diagnosis.

**Electronic supplementary material:**

The online version of this article (doi:10.1186/s12879-015-0938-4) contains supplementary material, which is available to authorized users.

## Background

Sepsis is a major cause of morbidity and mortality in all high income as well as middle and low income countries [1-5]. About 20 million cases of sepsis are estimated to occur each year around the world accounting for up to 135,000 deaths in Europe and 215,000 in the United States [1,4]. Recent studies from different countries show that the incidence of sepsis as well as the number of sepsis-related deaths is continuously increasing [3,6-11].

At present, blood culture is considered the gold standard for aetiological diagnosis in sepsis. Although blood culture is associated with high specificity in species identification, it is limited by a substantial time delay and low sensitivity, especially for slow-growing and fastidious organisms [12,13]. In an effort to address some of the limitations with blood culture, nucleic acid amplification tests (NAATs) have been developed to improve the sensitivity and decrease the time to identification of microorganisms causing bacteraemia. Several commercial multiplex assays, based on different techniques such as conventional polymerase chain reaction (PCR) (VYOO®, Analytik Gena, Germany), real-time PCR (Magicplex™ Sepsis Real-time Test, Seegene, Korea; LightCycler® SeptiFast, Roche, Switzerland; Sepsitest™, Molzym, Germany) and microarray (Prove-it™ Sepsis, Mobidiag, Finland) are now on the market. These assays have been tested in several studies [14-24] but rarely is there a clinical validation of the results.

The aim of this population-based study was therefore to evaluate the clinical utility of two commercial NAATs, Magicplex™ Sepsis Real-time Test and Prove-it™ Sepsis, in patients with suspected sepsis. Magicplex™ Sepsis Real-time Test is a PCR-based test screening for 73 species of Gram positive bacteria, twelve species of Gram negative, and six species of fungi and three resistance genes (*mecA*, *vanA*, and *vanB*) directly in whole blood samples. The microarray-based assay called Prove-it™ Sepsis combines genome amplification by conventional PCR and microarray technology for simultaneous identification of over 60 bacterial pathogens, and 13 fungal pathogens, and three resistance genes (*mecA*, *vanA*, and *vanB*) in positive blood cultures.

## Methods

### Patients and specimens

From September 2011 to June 2012, a prospective observational study of the incidence of community-onset severe sepsis and septic shock in adults was conducted at Skaraborg Hospital, in the western region of Sweden. During a limited period of the prospective study, from February to April 2012, NAATs were performed as part of routine patient care in addition to blood culture in all patients >18 years admitted to the emergency department for suspicion of community-onset sepsis. All patients received oral and written information about the study. In patients suspected to have sepsis or severe sepsis, it is mandatory to rapidly make appropriate sampling for microbiological diagnosis before antibiotic treatment is instituted. This microbiological testing, using appropriate and approved tests, needed no patient consent according to the Swedish Law. The diagnostic tests were made upon arrival to the emergency department and no additional sampling was made. Some patients were too sick and/or died within 24 hours and could never give an informed consent. Those patients who could not give an informed consent, were evaluated anonymously, only for diagnosis and results of commercially available tests used for diagnostic purposes of the suspected sepsis. The Regional Ethics Committee in Gothenburg (no. 376–11) approved the study and the consent process.

### Blood culture

In this study, an episode was defined as each separate case of clinically suspected community-onset sepsis treated with intravenous antibiotics. Only one episode per patient and admission were included in the final data analysis to avoid bias. For each episode, two sets of blood cultures from two different puncture sites were collected before administration of the first dose of intravenous antibiotics. However, for a few episodes, due to feasibility, only one set of blood culture was collected. Blood cultures were conducted in BacT/ALERT® FN (bioMérieux, France). Typing and definite species identification with MALDI-TOF MS was performed on a Microflex LT mass spectrometer (Bruker Daltonics, United States) with BioTyper software v2.0 using default parameter settings. Spectral scores above 2.0 were used as cut-off for correct identification. Antibiotic susceptibility was determined by accredited laboratory methods according to EUCAST guidelines (www.eucast.org).

### Multiplex PCR assay

The test procedure for Magicplex™ was performed on 1 mL fresh whole blood (EDTA) collected before administration of antibiotics and not older than 24 hours. DNA extraction from whole blood was performed using the SelectNA Blood Pathogen Kit (Molzym, Germany). The first steps of the extraction, lysis of human cells and digestion of human DNA, were manually performed according to the manufacturer’s instructions. Pure bacterial/fungal DNA was then extracted automatically from the prelysed samples on the instrument Nordiag Arrow/Liaison IXT (DiaSorin, Italy). The first PCR, producing amplicon banks, was performed using the kit Magicplex Sepsis Amplification on a GeneAmp PCR System 9700 (Applied Biosystems, United States). Real-time PCR was then performed with the Magicplex Screening Real-time Detection Kit on a CFX96 (Bio-Rad, United States). The screening revealed the presence of Gram positive or Gram negative bacteria, drug resistance genes or fungi. Species identification was performed with the Magicplex ID 1-ID 9 Real-time Detection Kit on samples that became positive in the screening step. All PCR-reactions were set up in a UV-box according to the recommendations from the manufacturer. The dedicated software Seegene viewer was used to interpret the analysis data, where the result from every sample is presented in a table as Detected or Not detected. A whole process control was included in the assay. If this was valid, the assay result could be interpreted.

### Microarray-based assay

Prove-it™ was performed on aliquots derived from blood culture bottles removed from the automated blood culture system. After removal, the bottles were stored in 4°C between 1–2 days until DNA extraction was performed. For each episode, aliquots were taken from that pair of blood culture bottles, one aerobic and one anaerobic bottle, derived from the first puncture site. A volume of 200 μL was withdrawn from each bottle. Regardless if the blood culture bottles were found positive or negative, sample volumes from each pair of bottles was pooled together prior to DNA extraction. DNA was extracted using 400 μL of sample volume and eluted in a final volume of 200 μL using a MagNA Pure Nucleic Acid Isolation Kit I on a MagNAPure Compact System (Roche Applied Science, Switzerland). The procedure for the microarray assay was performed according to protocols provided by the manufacturer. Briefly, both a bacterial and a fungal PCR master mix were prepared. The PCR reactions were set up in a laminar airflow bench where no amplified PCR products were handled. After PCR amplification, the bacterial PCR product and the fungal PCR product derived from the same episode were added to the same well of the microarray. Subsequently, hybridization and staining procedures were performed. For detection and analysis of the samples, the dedicated software Prove-it™ Advisor was used. Based on the outcomes of several built-in controls, the software evaluated whether the performance of the assay is acceptable. All analysis parameters were adjusted automatically without any manual involvement.

### Data interpretation and statistical analysis

All records of the patients and microbial findings were assessed by two senior physicians in infectious diseases (LL and GJ). The clinical judgment was based on the patient history with special reference to sudden onset of fever, rigors, gastrointestinal symptoms, tachypnea, mental confusion, pain out of proportion, and muscle weakness. Physical examination was done with attention to blood pressure <90 mm Hg, respiratory rate >20/min, and oxygen saturation <90%. Standard biomarkers included serum lactate, leucocyte cell count, neutrophil-lymphocyte count ratio, and C-reactive protein. Judgment was also based on imaging and microbiological testing of suspected infectious foci including culture, PCR, and antigen test, apart from the NAAT assays described in detail above.

**Figure 1:**
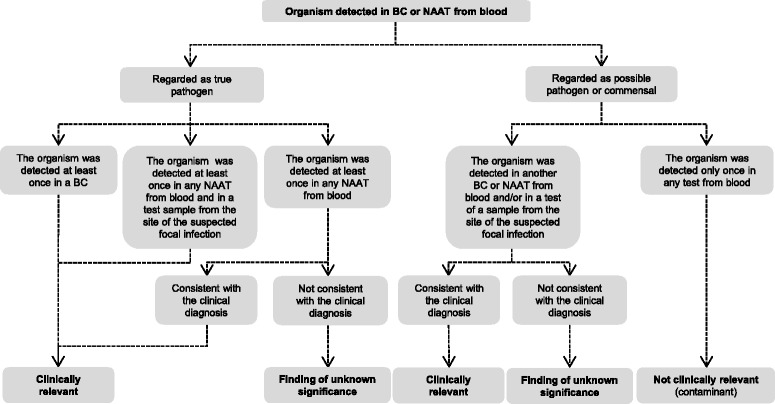
Algorithm for deciding on clinical relevance of microbial findings in blood by blood culture [25] or NAAT. Other cultures were made from clinically relevant sites before administration of intravenous antibiotics. On suspicion of pneumonia or sepsis with unknown focus, a pulmonary X-ray was performed. Ultrasound, computed tomography scan, and magnetic resonance imaging were used when deemed necessary for diagnosing the site of infection. *BC* blood culture; N*AAT* nucleic acid amplification test.

It remains challenging to determine the aetiological significance of organisms detected in blood. This applies especially to NAATs, but also to conventional blood culture although to a lesser degree. In this study, decisions of aetiological significance of detected organisms were made based on expected pathogenicity (Table 1) and clinical presentation as previously described [25,26]. Detected organisms were interpreted as clinically relevant, of unknown significance or contaminant according to the algorithm described in Figure 1. Organisms detected only by a NAAT were also regarded as clinically relevant, but with more stringent criteria than positive blood culture results. Microorganisms of unknown aetiological significance are findings considered not consistent with the clinical diagnosis having no implications for the medical management of the patients. However, links between the microorganism and the medical condition of the patient cannot be excluded.

**Table 1:** **Schematic distribution of microorganisms according to expected pathogenic potential**

**True pathogens**	**Possible pathogens**
*Bacteroides fragilis* group	*Aerococcus* spp.
*Candida* spp.	Anaerobic Gram positive cocci
*Clostridium* spp. other than *C. perfringens*	*Lactobacillus* spp.
*Enterobacteriaceae* spp.	Non-fermenting Gram negative rods
*Enterococcus* spp.	Viridans group streptococci
Former *Streptococcus bovis* group	
*Fusobacterium* spp.	
*Haemophilus* spp.	**Commensals (common contaminants)**
^a^HACEK group	
*Listeria* spp.	*Bacillus* spp.
*Mycobacterium* spp.	Coagulase-negative staphylococci
*Neisseria* spp.	*Corynebacterium* spp.
*Pasteurella* spp.	*Propionibacterium* spp.
*Porphyromonas* spp.	
*Prevotella* spp.	
*Pseudomonas aeruginosa*	
*Staphylococcus aureus*	
*Staphylococcus lugdunensis*	
*Stenotrophomonas maltophilia*	
*Streptococcus anginosus* group	
*Streptococcus* group A, B, C, G	
*Streptococcus pneumoniae*	

^a^HACEK group includes: *Haemophilus parainfluenzae, Haemophilus aphrophilus, Haemophilus paraphropilus, Actinobacillus actinomycetemcomitans, Aggregatibacter aphrophiuls, Cardiobacterium hominis, Eikenella corrodens, and Kingella kingae*.

McNemar’s test was conducted for comparing proportions in paired samples, whereas z test was performed for comparing proportions in independent samples. A two-sided *p* value of <0.05 was considered statistically significant. Concordances between blood culture and NAATs were tested with kappa statistics for inter-rater agreement; cut-off values for the kappa value have been described elsewhere; 0.41-0.60 are considered of moderate agreement, and those of 0.81-1.00 of very good agreement [27]. Statistical analyses were performed using Matlab v. 7.10 (The Mathworks, Inc., United States).

## Results

A total of 375 patients entered this study. Eight patients were admitted twice during the study period, resulting in a total of 383 episodes analysed with blood culture and both NAATs. For the multiplex PCR, the whole process control was invalid or partially invalid in 45 (12%) of the 383 blood samples initially tested. These samples were retested on a ten-fold dilution of the extracted DNA. After the re-run, one sample did still not give a final result and was excluded from further analysis, making a total of 382 episodes.

At least one microorganism was detected in 138 episodes by either method. Microorganisms defined as commensals (Table 1) were considered to be contaminants and excluded from analysis if only found on a single occasion regardless of detection method. In total, 89 clinically relevant findings or findings of unknown significance were identified in 77 episodes (20%). A detailed description including clinical comments for these findings can be found in Additional file 1. In eight episodes, a clinically relevant finding was detected by blood culture in a bottle not included in that pair of bottles from which aliquots for the microarray analysis was derived. Thus, these eight episodes were excluded from the analysis of the microarray results.

**Figure 2:**
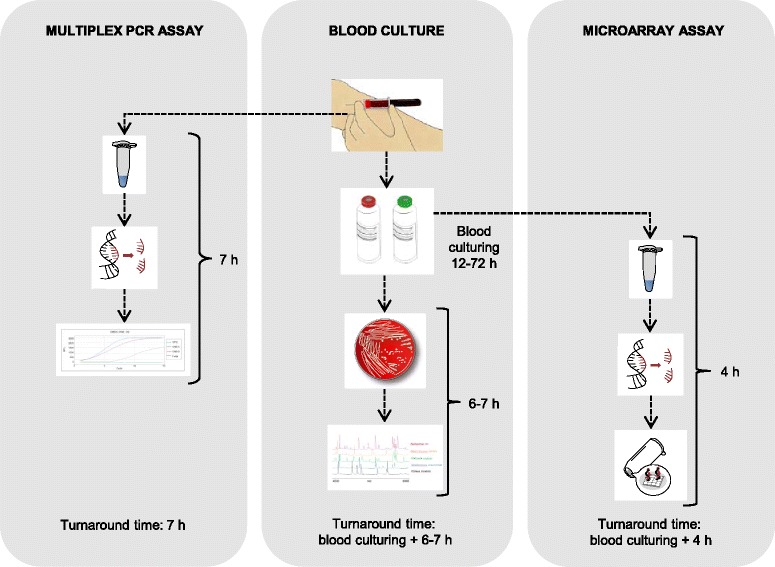
Workflows and turnaround times for the detection methods used in the present study.

For blood culture, the result of species identification was usually available 6–7 hours after a bottle has flagged positive (Figure 2). The turnaround time for the multiplex PCR assay was estimated to around seven hours. For the microarray, the turnaround time from positive blood culture bottle to species identification was approximately four hours. The diagnostic performance for blood culture and the NAATs are shown in Table 2.

**Table 2:** **Diagnostic performance of blood culture, multiplex PCR, and microarray** ^**a**^

	**Sensitivity**	**Specificity**	**PPV**	**NPV**	**Accuracy**
	**(95% CI)**	**(95% CI)**	**(95% CI)**	**(95% CI)**	**(95% CI)**
Blood culture	72% (61–84)	99% (99–100)	98% (93–100)	94% (92–97)	95% (92–97)
Multiplex PCR	64% (51–76)	96% (93–98)	73% (60–85)	94% (91–96)	91% (88–94)
Microarray	62% (49–75)	99% (97–100)	86% (75–97)	95% (92–97)	94% (91–96)

*NPV* negative predictive value; *PPV* positive predictive value.

^a^For each method, all episodes were classified according following criteria: i) true positive – episode positive for at least one clinically relevant finding; ii) false positive - episode positive only for finding(s) of unknown significance; iii) false negative - episode negative with the method, but positive for at least one clinically relevant finding detected by another method; iv) true negative - episode negative with the method and for which no clinically relevant findings were detected by any method.

### Multiplex PCR assay

The rate of episode positivity for multiplex PCR was 14% (53/382), whereas the rate of episode positivity for blood culture was 11% (42/382, *p* = 0.61). Cohen’s kappa coefficient for agreement between the results of multiplex PCR and blood culture was 0.52 (95% CI 0.37-0.66).

**Figure 3:**
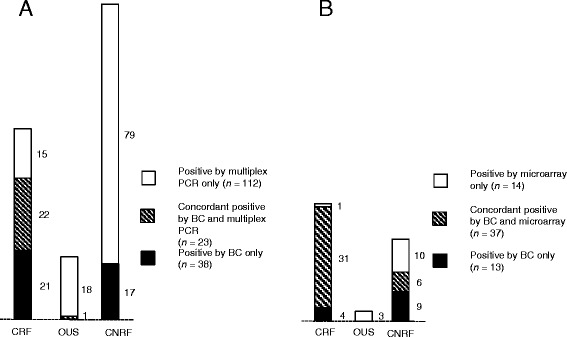
Microbial concordances between blood culture and NAATs. **A** Number of microbes detected by blood culture and/or multiplex PCR. **B** Number of microbes detected by blood culture and/or microarray. *BC* blood culture; *CRF* clinically relevant finding; *CNRF* clinically not relevant finding; *OUS* finding of unknown significance.

In total, 37 clinically relevant findings in 37 episodes and 23 findings of unknown significance in 21 episodes were detected by multiplex PCR. For blood culture, 44 findings in 42 episodes were clinically relevant and one finding of unknown significance. The multiplex PCR and blood culture results agreed for 22 clinically relevant findings (Figure 3A). Multiplex PCR missed 22 clinically relevant findings in 20 episodes and one finding of unknown significance. On the other hand, 38 findings identified by multiplex PCR in 35 episodes were not detected by blood culture. Fifteen of these 38 findings were considered as clinically relevant; five of these 15 findings were considered as proven aetiology of the infections since the same bacteria were found by culture from the site of infection (Table 3). The remaining ten findings made by multiplex PCR were negative in blood culture and other diagnostic tests, but consistent with the clinical diagnosis and therefore regarded as clinically relevant (Table 3). Twenty-three of the 38 findings detected by multiplex PCR but not by blood culture were regarded to be of unknown significance. Ten were findings of Gram positive bacteria, eleven were findings of Gram negative bacteria and two were findings of *Candida* species (Table 4).

**Table 3:** **Clinically relevant microbial findings made by multiplex PCR but not by blood culture**

**Patient**	**Multiplex PCR results**	**Clinical diagnosis**
1	*Enterobacter aerogenes*	Abdominal abscess.
2	*Enterococcus faecalis* ^a^	Acute pyelonephritis. Grew *E. faecalis* in the urine.
3	*Escherichia coli* ^a^	Acute pyelonephritis. Grew *E. faecalis* in the urine.
4	*Escherichia coli* ^a^	Acute pyelonephritis. Grew *E. faecalis* in the urine.
5	*Escherichia coli* ^a^	Acute pyelonephritis. Grew *E. faecalis* in the urine.
6	*Escherichia coli*	Acute cholecystitis. Elevated liver enzymes and positive computed tomography scan. Three months earlier, the patient had an acute cholecystitis with *E. coli* in blood cultures.
7	*Escherichia coli*	Acute pyelonephritis. Urine culture could not be obtained.
8	*Escherichia coli*	Abdominal abscess after appendectomy.
9	*Klebsiella oxytoca*	Perianal abscess.
10	*Klebsiella pneumoniae*	Acute pyelonephritis. Urine culture could not be obtained.
11	*Staphylococcus aureus* ^a^	Infected leg ulcer. Grew *S. aureus* in the ulcer.
12	*Stenotrophomonas maltophilia*	Infected chronic leg ulcer.
13	*Streptococcus pneumoniae*	Clinical pneumonia, confirmed by chest x-ray.
14	*Streptococcus pneumoniae*	Fever, respiratory distress, pulmonary fibrosis. No other diagnosis was made. Chest x-ray inconclusive for pneumonia.
15	*Streptococcus* spp.	Acute cholangitis. Elevated liver enzymes and positive computed tomography scan.

^a^Considered as proven aetiology since the same species were found by culture from the site of infection.

**Table 4:** **Microbial findings of unknown significance made by multiplex PCR but not by blood culture**

**Patient**	**Multiplex PCR results**	**Clinical diagnosis**
1	*Acinetobacter baumannii*	Acute pyelonephritis due to *E. coli*.
2	*Acinetobacter baumannii*	Infected chronic leg ulcer. *A. baumannii* not cultured from wound.
3^a^	*Acinetobacter baumannii, Stenotrophomonas maltophilia*	Infected chronic leg ulcers.
4	*Acinetobacter baumannii*	Pulmonary embolism.
5	*Candida krusei*	Pneumonia verified by chest X-ray.
6	*Candida parapsilosis*	Acute pyelonephritis due to *E. coli*.
7	*Enterobacter cloacae*	Polycytemia vera. Fever and respiratory distress. Believed to have an adverse reaction to hydroxyurea. Infiltrate on chest X-ray. PCR from nasopharynx positive for human metapneumovirus and coronavirus.
8	*Enterococcus faecalis*	Acute pyelonephritis due to *E. coli*.
9	*Enterococcus gallinarium*	Nausea. No other diagnosis was made.
10	*Escherichia coli*	Acute otitis media. No other cultures were positive.
11	*Klebsiella oxytoca*	*S. aureus* endocarditis.
12	*Klebsiella pneumoniae*	Acute vomiting and fever. No other diagnosis was made.
13	*Pseudomonas aeruginosa*	Acute pyelonephritis due to *E. coli*.
14^a^	*Staphylococcus aureus, Streptococcus* spp.	Influenza A verified by PCR.
15	*Staphylococcus aureus*	Pneumonia due to *K. pneumoniae*. Lymphoma.
16	*Staphylococcus aureus*	Pneumonia. Verified by chest X-ray.
17	*Stenotrophomonas maltophilia*	Pulmonary fibrosis and suspected pneumonia.
18	*Streptococcus agalactiae*	Arrived with severe sepsis. Diseased within 24 hours.
19	*Streptococcus pneumoniae*	Acute diabetic ketoacidosis. PCR from nasopharynx positive for human rhinovirus. Highly elevated neutrophil count.
20	*Streptococcus pneumoniae*	Acute pyelonephritis due to *E. coli*.
21	*Streptococcus* spp.	Goiter.

^a^Two microbial findings of unknown significance in the same patient.

### Microarray-based assay

The rate of episode positivity for the microarray assay was 8.4% (32/382) and for blood culture 8.9% (34/382, *p* = 0.37). Cohen kappa statistic for agreement between the results of microarray and blood culture was 0.92 (95% CI 0.84-0.99).

In total, 32 clinically relevant findings in 31 episodes and five findings of unknown significance in five episodes were detected by microarray. The results between microarray and blood culture showed concordance for 30 clinically relevant findings (Figure 3B). The microarray failed to detect four clinically relevant findings detected by blood culture. However, two of these findings belonged to species not covered by the microarray panel (*Streptococcus anginosus* and *Streptococcus* group G). The microarray identified one clinically relevant finding and five findings of unknown significance not detected by either blood culture or multiplex PCR (Table 5).

**Table 5:** **Microbial findings made by microarray but not by blood culture**

**Patient**	**Microarray results**	**Clinical diagnosis**
1	*Enterobacter cloacae* ^b^	Tendinitis of the hand due to *S. aureus*.
2	*Enterobacter cloacae* ^b^	Acute pyelonephritis due to *E. faecalis.*
3	*Enterobacter cloacae* ^b^	Neutropenic fever.
4	*Enterococcus faecalis* ^*a*^	Acute bronchitis. *E. faecalis* in urine.
5	*Staphylococcus aureus* ^b^	Pneumonia.
6	*Staphylococcus aureus* ^b^	Acute pyelonephritis due to *E. coli.*

^a^Clinically relevant microbial finding.

^b^Microbial finding of unknown significance.

## Discussion

The focus of this study is on the clinical utility of two commercial NAATs in the aetiological diagnosis of patients with suspected sepsis. We found that the multiplex PCR assay added diagnostic value by timely detection of clinically relevant microbes missed by blood culture. The results of the microarray-based assay corresponded well to those of blood culture, but its clinical utility is reduced by the prerequisite of time-consuming cultivation.

Currently, blood culture is considered the gold standard for aetiological diagnosis of sepsis although it suffers from having a low sensitivity. It can only detect viable microorganisms, and the medium is not optimized for culturing fungi and fastidious bacteria. Thus, blood culture can be considered to be a poor gold standard, which implies difficulties in evaluating novel sepsis tests since no other laboratory reference standard for sepsis diagnosis exists. For that reason, different reference standards have been used in studies assessing sepsis tests. Some have used blood culture results alone as gold standard [20,28], whereas others have considered all pathogenic findings detected by any method [16,29]. We assessed the performances of blood culture and the two NAATs by classifying all findings judged as clinically relevant as “true positives”. The diagnostic sensitivity for multiplex PCR (64%, Table 2) was comparable with the rate in a similar study by Carrara *et al*. (65%, *p* = 0.91) [16], as well as the specificity (96% vs. 92%, *p* = 0.05) [16]. However, Loonen *et al*. have reported significantly lower sensitivity (37%, *p* < 0.001) and specificity (77%, *p* = 0.0001) for Magicplex™ [19]. For the microarray assay, we observed a significantly lower diagnostic sensitivity (62% vs. 96%, *p* < 0.0001, Table 2) whereas the specificity was equal (99% vs. 99%, *p* = 1.0) compared with a previous study [20]. The use of different gold standards and study populations may explain observed differences in the performance characteristics [16,19,20,30].

Although the multiplex PCR assay could not detect all clinically relevant microbes, it offered added diagnostic value by the detection of several important pathogens not detected by the conventional culture-based method (Table 3). Consequently, the concordance between the results of blood culture and multiplex PCR assay is moderate (kappa 0.50) (Table 2), whereas the microarray results showed a high degree of agreement with the results of blood culture (kappa 0.90) (Table 2). These results could be expected since the microarray assay is performed on aliquots derived from blood culture bottles, whereas the multiplex PCR is performed on microbial DNA extracted from a different whole blood sample than the cultured portion.

A remarkable finding is that multiplex PCR of only 1 mL whole blood reached a sensitivity of 64% compared to 72% for culture using 32–40 mL blood, despite more stringent criteria for clinical relevance in the PCR case. However, both methods failed to detect a number of sepsis cases. From a strictly quantitative aspect, the detection level for microorganisms in blood samples at a given time, the diagnostic sensitivity, is depending on the concentration of microorganisms and the volume sampled. The extent of variation in the yield of bacteria in time is not known. In bacteraemia, an average concentration of 0.25 CFU/mL blood was reported by Arpi *et al*. [31]. Jonsson *et al*. [32] theoretically calculated the probability of detecting bacteria as a function of the concentration in blood and found empirically by blood culture that 29% of all cases with *Escherichia coli* and 18% of *S. aureus* bacteraemia had a most probable concentration of only 0.036 CFU/mL. The gain in yield of microorganisms by increasing the volume of cultured blood in more modern automated culture systems was emphasized by Cockerhill *et al*. [33] and Lee *et al.* [34]. To obtain a >99% sensitivity with these systems, four blood cultures, each involving 20 mL is needed [34]. The gain is probably due to the detection of the most minute concentrations, either by enhanced culture systems or overcoming the effects of early antibiotic therapy by resins.

NAATs introduced to diagnose sepsis by sampling blood may add new information implying higher analytical sensitivity. By definition, the analytical sensitivity of an assay refers to the smallest value of the analyte that can be resolved with a given degree of confidence and is not synonymous with the diagnostic sensitivity. A single bacterial cell is obviously the smallest theoretical unit that can be applied for blood culture or culture in a wider sense. With NAATs, a single bacterial cell, dead or alive, may contain multiples of a certain target sequence, e.g., in bacteria the conserved 16S rRNA gene sequence is often used as PCR template due to its high copy number in each cell. The downside of using detection methods with high analytical sensitivity, such as PCR, is the increased number of findings of unknown significance as well as contaminants. We then have to cope with unexpected microbial findings that do not necessarily corroborate with the clinical picture, as translocation of bacteria and fungi over the mucosal gut barrier in patients with malignancies or on parenteral nutrition is an increasing diagnostic dilemma. Thus, we are urged to tighten the communication between the laboratory and the clinicians to organise, assemble and critically review the unexpected findings.

There is also a qualitative aspect. Certain species seem to correlate better with genuine sepsis than others, i.e., *Enterobacteriaceae* spp. and pneumococci [35]. The detection of identical microorganisms from multiple sampling sites or occasions is another genuine marker for true bacteraemia. This further implies that a single finding of a microbe with one detection system, e.g., culture, may move the interpretation from probable to proven if the same finding is done with e.g., PCR. However, true bacteraemia also occurs in patients void of an inflammatory response. This shall not be regarded as a benign finding until the following factors are ruled out. First, translocation of bacteria from the gut to the bloodstream may occur in patients with malfunctioning mucosal barriers. Well known examples are *E. cloacae* (occult malignancy) [35], *Streptococcus* group G, i.e., *Streptococcus dysgalactiae* subsp. *equisimilis* (haematological malignancy and solid tumours) [36], and the former *Streptococcus* bovis group, i.e., *S. gallolyticus* subsp. *gallolyticus* and subsp. *pasteurianus* (colonic cancer), and *S. infantarius* (cancer of the bile tree or pancreas) [37]. Experimental work on animals showed an increased risk for bacterial translocation for subjects fed exclusively by the parenteral route [38]. Finally, there are a large number of conditions linked to immunodeficiencies where both mucosal barriers and normal inflammatory response are malfunctioning. A significant microbial finding, e.g., single detection of a species with significant pathogenic profile or multiple detection of the same but low-pathogenic species, should alert the clinician independent of clinical signs of sepsis and whether the detection method was culture or molecular. Combining the methods might therefore provide important clinical information concerning not only the acute infection but also underlying conditions.

In our laboratory, it usually takes around 6–7 hours after a blood culture bottle has flagged positive before the isolate has grown enough on the plates enabling species identification within minutes by MALDI-TOF MS (Figure 2). At the same time a primary reading of the antibiogram is done and aids to disclose resistant strains of *Staphylococcus aureus, Enterobacteriaceae spp*. and non-fermenters. However, the time needed for species identification by conventional culture-based methods differs between clinical laboratories depending on routines. For the microarray, the turnaround time from positive bottle to microorganism identification was about four hours including preparation of blood culture bottles and DNA extraction. We estimated that the use of the microarray would save 2–3 hours compared to routine methods, but it is more labour intensive. For both blood culture and microarray, the incubation time of typically 1–3 days must also be considered. However, a time saving was obtained using multiplex PCR with an estimated turnaround time of only seven hours since this assay was performed directly on whole blood sample requiring no incubation time.

For the multiplex PCR assay, the whole process control was invalid or partially invalid in 45 (12%) of the 383 blood samples initially tested. According to the manufacturer, the whole process control indicates whether the process has functioned optimally or not. No further information is given when the whole process control is flagged as invalid. We speculated that a very high DNA concentration in the samples either inhibited the PCR reaction or the purification process. Therefore, these samples were retested on a ten-fold dilution of the extracted DNA and all but one sample then gave a final result. The high rate of invalid whole process control thus reduces the clinical utility of multiplex PCR since such samples need to be retested.

Our study has several limitations. One of the most important is that TATs were not precisely measured, just roughly estimated, mainly due to handling procedures. In addition, none of the NAATs were run 24/7; multiplex PCR was performed once daily whereas the microarray was ran every second day.

## Conclusions

Our results indicate that the use of multiplex PCR assays on whole blood specimens may shorten the time to identifying causative agents in blood. As for the microarray-based assay, it corresponds well to blood culture, but the prerequisite of time-consuming cultivation reduces its clinical utility. Most important, we found that the multiplex PCR assay has the potential to serve as a complement to blood culture in detecting clinically relevant microbes in blood. However, it should be stressed that the aetiological significance of findings detected by PCR-based assays should be interpreted with caution as the high analytical sensitivity may add findings that do not necessarily corroborate with the clinical diagnosis. Improving PCR technology, e.g., repeated sampling or increasing diagnostic sensitivity by processing a larger volume of blood, by faster and automated analysis will make NAATs a better tool for diagnosing septic patients.

## Additional file

Additional file 1:
**All 77 episodes for which at least one microbial finding judged as clinically relevant or of unknown significance was detected with blood culture and/or NAATs.** Table with all microbial findings judged as clinically relevant or of unknown significance detected by blood culture and/or NAATs.

## Abbreviations

NAAT: nucleic acid amplification test
NPV: negative predictive value
PCR: polymerase chain reaction
PPV: positive predictive value
