# Supplementary material for: Clinical evaluation of commercial nucleic acid amplification tests in patients with suspected sepsis
Source: BMC Infect Dis. 2015 Apr 28;15:199. doi: 10.1186/s12879-015-0938-4 (PMC4419503; doi:10.1186/s12879-015-0938-4)
Supplement: Additional file 1: — All 77 episodes for which at least one microbial finding judged as clinically relevant or of unknown significance was detected with blood culture and/or NAATs. Table with all microbial findings judged as clinically relevant or of unknown significance detected by blood culture and/or NAATs. [file 12879_2015_938_MOESM1_ESM.pdf]

Additional file 1. All 77 episodes for which at least one microbial finding judged as clinically relevant or of unknown significance was detected with blood culture and/or NAATs.

**No.**	**Blood culture results (proportion positive blood culture bottles)**	**Other microbiological findings**	**Multiplex PCR results**	**Microarray results**	**Clinical comments**
1	*Escherichia coli* (1/4)[a]	-	neg	neg	*E. coli* is clinically relevant. Diagnosis: acute pyelonephritis due to *E. coli*. No SS. LoS 8 days.
2	*Enterococcus faecalis* (1/4)[a]	Urine*: Enterococcus faecalis*	neg	neg	*E. faecalis is* clinically relevant. Diagnosis: acute pyelonephritis due to *E. faecalis.* No SS. LoS 7 days.
3	*Enterococcus faecalis* (1/4)[a]	Urine: *Enterococcus faecalis*	neg	neg	*E. faecalis* is clinically relevant. Diagnosis: acute pyelonephritis due to *E. faecalis*. No SS. LoS 8 days.
4	*Escherichia coli* (2/2)	Urine: *Escherichia coli*	neg	*Escherichia coli*	*E. coli* is clinically relevant. Diagnosis: acute pyelonephritis due to *E. coli*. No SS. LoS 5 days.
5	*Escherichia coli* (2/2)[a]	Urine*: Escherichia coli*	neg	neg	*E. coli* is clinically relevant. Diagnosis: acute pyelonephritis due to *E. coli*. No SS. LoS 10 days.
6	*Escherichia coli* (2/4)	Urine: *Escherichia coli*	*Escherichia coli, Enterococcus faecalis*	*Escherichia coli*	*E. coli* is clinically relevant, *E. faecalis* is OUS. Diagnosis: acute pyelonephritis. No SS. LoS 1 day.
7	*Escherichia coli* (2/4)	Urine: *Escherichia coli*	*Escherichia coli*	*Escherichia coli*	*E. coli* is clinically relevant. Diagnosis: acute pyelonephritis due to *E. coli*. No SS. LoS 8 days.
8	*Escherichia coli* (2/4)[a]	-	neg	neg	*E. coli* is clinically relevant. Diagnosis: acute cholecystitis due to *E. coli*. No SS. LoS 2 days.
9	*Escherichia coli* (3/4)	Urine*: Escherichia coli*	*Escherichia coli* , *Pseudomonas aeruginosa*	*Escherichia coli*	*E. coli* is clinically relevant, *P. aeruginosa* is OUS. Diagnosis: acute pyelonephritis due to *E. coli*. No SS. LoS 1 day.
10	*Escherichia coli* (3/4)	-	*Escherichia coli*	*Escherichia coli*	*E. coli* is clinically relevant. Diagnosis: acute cholecystitis. No SS. LoS 16 days.
11	*Escherichia coli* (3/4)	-	neg	*Escherichia coli*	*E. coli* is clinically relevant. Diagnosis: acute pyelonephritis due to E. coli. No SS. LoS 3 days.
12	*Escherichia coli* (4/4)	-	*Escherichia coli*	*Escherichia coli*	*E. coli* is clinically relevant. Diagnosis: acute cholangitis due to *E. coli*. No SS. LoS 3 days.
13	*Escherichia coli* (2/2), *Klebsiella pneumoniae* (2/2)[a]	-	*Escherichia coli*	*Escherichia coli*	*E. coli* and *K. pneumoniae* are clinically relevant. Diagnosis: acute cholangitis due to *E. coli* and *K. pneumoniae*. SS. LoS 10 days.
14	*Escherichia coli* (2/4), *Klebsiella pneumoniae* (2/4)	-	neg	*Escherichia coli, Klebsiella pneumoniae*	*E. coli* and *K. pneumoniae* are clinically relevant. Diagnosis: acute cholangitis. SS. LoS 12 days.
15	*Klebsiella oxytoca* (4/4)	Urine: *Klebsiella oxytoca*	neg	*Klebsiella oxytoca*	*K. oxytoca* is clinically relevant. Diagnosis: acute pyelonephritis due to *K. oxytoca*. No SS. LoS 6 days.

16	*Klebsiella pneumoniae* (1/4)[a]	Urine: *Escherichia coli*	*Staphylococcus aureus*	neg	*K. pneumoniae* is clinically relevant, *S. aureus* is OUS. Diagnosis: lymphoma and pneumonia due to *K. pneumoniae*. SS. LoS 26 days.
17	*Klebsiella pneumoniae* (4/4)	NPH: *Streptococcus* group G; urine: *Klebsiella pneumoniae*	*Klebsiella pneumoniae*	*Klebsiella pneumoniae*	*K. pneumoniae* is clinically relevant. Diagnosis: acute cholangitis due to *K. pneumoniae*. SS. LoS 26 days. Gall bladder cancer.
18	*Klebsiella pneumoniae* (4/4)	NPH, urine: *Klebsiella pneumoniae*	neg	*Klebsiella pneumoniae*	*K. pneumoniae* is clinically relevant. Diagnosis: acute pyelonephritis due to *K. pneumoniae*. No SS. LoS 4 days.
19	*Pseudomonas aeruginosa* (1/4)	-	neg	*Pseudomonas aeruginosa*	*P. aeruginosa* is clinically relevant. Diagnosis: cellulitis due to *P. aeruginosa*. No SS. LoS 12 days.
20	*Proteus mirabilis* (4/4)	Urine: *Proteus mirabilis*	*Proteus mirabilis*	*Proteus mirabilis*	*P. mirabilis* is clinically relevant. Diagnosis: acute pyelonephritis due to *P. mirabilis.* SS. LoS 6 days.
21	*Staphylococcus aureus* (1/4)	-	*Staphylococcus aureus*	*Staphylococcus aureus*	*S. aureus* is clinically relevant. Diagnosis: *S. aureus* bacteraemia. Septic shock. LoS 1 day. Diseased.
22	*Staphylococcus aureus* (2/4)	Urine: *Staphylococcus aureus;* wound: *Staphylococcus aureus, Escherichia coli*	neg	*Staphylococcus aureus*	*S. aureus* is clinically relevant. Diagnosis: post-operative wound infection. No SS. LoS 16 days.
23	*Staphylococcus aureus* (3/4)	Wound: *Staphylococcus aureus*	*Staphylococcus aureus*	*Staphylococcus aureus*	*S. aureus* is clinically relevant. Diagnosis: skin infection. Abscess left leg. No SS. LoS 5 days.
24	*Staphylococcus aureus* (3/4)	-	neg	*Staphylococcus aureus*	*S. aureus* is clinically relevant. Diagnosis: *S. aureus* bacteraemia. No SS. LoS 7 days.
25	*Staphylococcus aureus* (4/4)	-	*Staphylococcus aureus*	*Staphylococcus aureus*	*S. aureus* is clinically relevant. Diagnosis: clinical central line infection. Severe sepsis. LoS 4 days.
26	*Staphylococcus aureus* (4/4)	-	*Staphylococcus aureus*	*Staphylococcus aureus*	*S. aureus* is clinically relevant. Diagnosis: *S. aureus* bacteraemia, spondylodiscitis, epidural abscess. No SS. LoS 46 days.
27	*Staphylococcus aureus* (4/4)	Urine: *Enterococcus faecalis*	*Staphylococcus aureus*	*Staphylococcus aureus*	*S. aureus* is clinically relevant. Diagnosis: *S. aureus* bacteraemia and SS. Los 6 days. Diseased.
28	*Staphylococcus aureus* (4/4)	Urine: *Escherichia coli*	neg	*Staphylococcus aureus*	*S. aureus* is clinically relevant. Diagnosis: *S. aureus* bacteraemia. No SS. LoS 12 days.
29	*Staphylococcus aureus* (4/4)	Urine: *Escherichia coli* , *Staphylococcus aureus*; wound: *Staphylococcus aureus*	*Klebsiella oxytoca*, *Staphylococcus aureus*	*Staphylococcus aureus*	*S. aureus* is clinically relevant. *K. oxytoca* is OUS. Diagnosis: *S. aureus* endocarditis. No SS. LoS 28 days.
30	*Staphylococcus aureus* (4/4)	NPH, wound: *Staphylococcus aureus*	*Staphylococcus aureus*	*Staphylococcus aureus*	*S. aureus* is clinically relevant. Diagnosis: acute post-operative mediastinitis. No SS. LoS 10 days.
31	*Staphylococcus aureus* (4/4)	-	neg	*Staphylococcus aureus*	*S. aureus* is clinically relevant. Diagnosis: *S. aureus* bacteraemia. No SS. LoS 17 days. Diseased.
32	neg	-	*Streptococcus agalactiae*	neg	*S. agalactiae* is OUS. SS. LoS 1 day. Diseased.

33	*Staphylococcus epidermidis* (4/4)	NPH: *Streptococcus pneumoniae*	*Staphylococcus epidermidis*	*Staphylococcus epidermidis*	*S. epidermidis* is clinically relevant. Diagnosis: central line infection due to *S. epidermidis*. No SS. LoS 6 days.
34	*Streptococcus anginosus (2/4)*[c]	-	*neg*	neg	*S. anginosus* is clinically relevant. Diagnosis: sub-acute endocarditis. No SS. LoS 26 days.
35	*Streptococcus* group G *(1/4)*[c]	-	*neg*	neg	*Streptococcus* group G is clinically relevant. Diagnosis: soft tissue infection left leg due to *Streptococcus* group G. SS. LoS 26 days.
36	*Streptococcus mitis* (1/4)	NPH: *Staphylococcus aureus*	neg	neg	*S. mitis* is clinically relevant. Diagnosis: streptococcal skin infection. No SS. LoS 6 days.
37	*Streptococcus parasanguinis* (1/4)	NPH: influenza A	neg	neg	*S. parasanguinis* is OUS. Diagnosis: influenza A, verified by PCR. No SS. LoS 5 days.
38	*Streptococcus pneumoniae* (1/2)	Urine: alpha streptococci, *Escherichia coli* , *Klebsiella pneumoniae*	*Streptococcus pneumoniae*	*Streptococcus pneumoniae*	*S. pneumoniae* is clinically relevant. Diagnosis: pneumonia due to *S. pneumoniae*. SS. LoS 5 days. Diseased.
39	*Streptococcus pneumoniae* (1/4)[a]	-	neg	neg	*S. pneumoniae* is clinically relevant. Diagnosis: pneumonia. No SS. LoS 0 days.
40	*Streptococcus pneumoniae* (4/4)	-	*Streptococcus pneumoniae*	neg	*S. pneumoniae* is clinically relevant. Diagnosis: pneumonia due to *S. pneumoniae*. No SS. LoS 3 days.
41	*Streptococcus pneumoniae* (4/4)	NPH: *Streptococcus pneumoniae*	*Streptococcus pneumoniae*	*Streptococcus pneumoniae*	*S. pneumoniae* is clinically relevant. Diagnosis: pneumonia due to *S. pneumoniae*. SS. LoS 9 days.
42	*Streptococcus pneumoniae* (4/4)	NPH: influenza A, *Streptococcus pneumoniae*; urine: *Escherichia coli*	*Streptococcus pneumoniae*	*Streptococcus pneumoniae*	*S. pneumoniae* is clinically relevant. Diagnosis: pneumonia due to *S. pneumoniae*. No SS. LoS 6 days.
43	*Streptococcus pyogenes* (2/2)	-	*Streptococcus pyogenes*	neg	*S. pyogenes* is clinically relevant. Diagnosis: septicaemia due to *S. pyogenes*. SS. LoS 5 days.
44	*Streptococcus pyogenes* (4/4)	NPH, wound: *Streptococcus pyogenes*	*Streptococcus pyogenes*	*Streptococcus pyogenes*	*S. pyogenes* is clinically relevant. Diagnosis: necrotising fasciitis due to *S. pyogenes*. Septic shock. LoS 27 days. Diseased.
45	neg	Wound: *Staphylococcus aureus*	*Acinetobacter baumannii*	neg	*A. baumannii* is OUS. Diagnosis: infected diabetic leg ulcer. No SS. LoS 7 days.
46	neg	Urine: *Escherichia coli*	*Acinetobacter baumannii*	neg	*A. baumannii* is OUS. Diagnosis: *E. coli* pyelonephritis. No SS. LoS 3 days.
47	neg	-	*Acinetobacter baumannii*	neg	*A. baumannii* is OUS. Diagnosis: pulmonary embolism. No SS. LoS 2 days.
48	neg	Urine: *Escherichia coli*	*Acinetobacter baumannii, Stenotrophomonas maltophilia*	neg	*A. baumannii* and *S. maltophilia* are OUS. Diagnosis: chronic leg ulcer and acute soft tissue infection. Cured by oxacillin. No SS. LoS 21 days.
49	neg	-	*Candida krusei*	neg	*C. krusei* is OUS. Diagnosis: pneumonia verified by X-ray. No SS. LoS 20 days.
50	neg	Urine: *Escherichia coli*	*Candida parapsilosis*	neg	*C. parapsilosis* is OUS. Diagnosis: pyelonephritis due to *E. coli*. No SS. LoS 7 days.

51	neg	Urine: *Candida albicans*	*Enterobacter aerogenes*	neg	*E. aerogenes* is clinically relevant. Diagnosis: abdominal abscess. No SS. LoS 34 days.
52	neg	NPH: coronavirus, human metapneumovirus	*Enterobacter cloacae*	neg	*E. cloacae* is OUS. Diagnosis: adverse reaction to hydroxyurea. No infection. No SS. LoS 6 days.
53	neg	Urine: *Enterococcus faecalis*	*Enterococcus faecalis*	*Enterobacter cloacae*	*E. faecalis* is clinically relevant. *E. cloacae* is OUS. Diagnosis: *E. faecalis* pyelonephritis. No SS. LoS 12 days.
54	neg	-	*Enterococcus gallinarum*	neg	*E. gallinarium* is OUS. Diagnosis: nausea. No SS. LoS 2 days. Received 1 dose of cefotaxime.
55	neg	-	*Escherichia coli*	*Staphylococcus aureus*	*E. coli* is clinically relevant. *S. aureus* is OUS. Diagnosis: acute pyelonephritis. No urine culture. No SS. LoS 4 days.
56	neg	-	*Escherichia coli*	neg	*E. coli* is clinically relevant. Diagnosis: acute cholecystitis. No SS. LoS 12 days. In Nov. 2011 acute cholecystitis with *E. coli* bacteraemia and septic shock.
57	neg	-	*Escherichia coli*	neg	*E. coli* is clinically relevant. Diagnosis: abscess after appendectomy. No SS. LoS 5 days.
58	neg	Urine: *Escherichia coli*	*Escherichia coli*	neg	*E. coli* is clinically relevant. Diagnosis: acute pyelonephritis due to *E. coli*. No SS. LoS 1 day.
59	neg	Urine: *Escherichia coli*	*Escherichia coli*	neg	*E. coli* is clinically relevant. Diagnosis: *E. coli* pyelonephritis. No SS. LoS 4 days.
60	neg	-	*Escherichia coli*	neg	*E. coli* is OUS. Diagnosis: acute purulent media otitis. No SS. LoS 5 days.
61	neg	Urine: *Escherichia coli*	*Escherichia coli* , *Streptococcus pneumoniae*	neg	*E. coli* is clinically relevant. *S. pneumoniae* is OUS. Diagnosis: acute pyelonephritis due to *E. coli*. No SS. LoS 2 days.
62	neg	Urine, wound: *Escherichia coli*	*Klebsiella oxytoca*	neg	*K. oxytoca* is clinically relevant. Diagnosis: perianal abscess. No SS. LoS 8 days.
63	neg	-	*Klebsiella pneumoniae*	neg	*K. pneumoniae* is OUS. Vomiting. Pneumonia? UTI? CRP max is 66. No SS. LoS 17 days.
64	neg	-	*Klebsiella pneumoniae*	neg	*K. pneumoniae* is clinically relevant. Diagnosis: acute pyelonephritis. Culture negative. No SS. LoS 2 days.
65	neg	NPH: *Moraxella catarrhalis;* urine: mixed flora; wound: *Staphylococcus aureus*	*Staphylococcus aureus*	neg	*S. aureus* is clinically relevant. Diagnosis: infected foot ulcer and pneumonia. No SS. LoS 12 days.
66	neg	-	*Staphylococcus aureus*	neg	*S. aureus* is OUS. Diagnosis: pneumonia. No SS. LoS 1 day.
67	neg	NPH: influenza A; urine: mixed flora	*Staphylococcus aureus, Streptococcus* spp.[b]	neg	*S. aureus* and *Streptococcus* spp. are OUS. Diagnosis: influenza A verified by PCR. 4 doses of PCG. No SS. LoS 6 days.

68	neg	-	*Streptococcus pneumoniae*	neg	*S. pneumoniae* is clinically relevant. Diagnosis: pneumonia and SS. LoS 5 days.
69	neg	-	*Stenotrophomonas maltophilia*	neg	*S. maltophilia* is clinically relevant. Diagnosis: infected chronic leg ulcers. No SS. LoS 23 days.
70	neg	-	*Stenotrophomonas maltophilia*, *Streptococcus pneumoniae*	neg	*S. maltophilia* is OUS, *S. pneumoniae* is clinically relevant. Diagnosis: pulmonary fibrosis, suspected pneumonia. No SS. LoS 10 days.
71	neg	NPH: human rhinovirus	*Streptococcus pneumoniae*	neg	*S. pneumoniae* is OUS. Diagnosis: diabetic ketoacidosic. No clinical infection. No SS. LoS 3 days.
72	neg	-	*Streptococcus* spp.[b]	neg	*Streptococcus* spp. is OUS. Diagnosis: goiter. No antibiotic treatment. LoS 3 days.
73	neg	-	*Streptococcus* spp.[b]	neg	*Streptococcus* spp. is clinically relevant. Diagnosis: acute cholangitis. No SS. LoS 11 days.
74	neg	-	neg	*Enterobacter cloacae*	*E. cloacae* is OUS. Diagnosis: neutropenic fever. No SS. LoS 5 days.
75	neg	Wound: *Staphylococcus aureus*	neg	*Enterobacter cloacae*	*E. cloacae* is OUS. Diagnosis: tendinitis of the hand due to *S. aureus*. No SS. LoS 7 days.
76	neg	Urine: *Enterococcus faecalis*	neg	*Enterococcus faecalis*	*E. faecalis* is clinically relevant. Diagnosis: acute bronchitis (probably viral). No SS. LoS 2 days. Received one dose of tobramycin and 2 doses of penicillin G that would be effective against enterococci.
77	neg	-	neg	*Staphylococcus aureus*	*S. aureus* is OUS. Diagnosis: pneumonia. No SS. LoS 4 days.

LoS length of stay; *neg* no microbial finding was detected; *NAAT* nucleic acid amplification test; *NPH* nasopharynx; *OUS* of unknown significance; *SS* severe sepsis.

[a] Microbial findings were detected in blood culture bottle(s) not included in that pair of bottles used for sampling blood material for microarray analysis.

[b] Further speciation not provided.

[c] Species not included in the microarray panel.
